# Development of frontoparietal connectivity predicts longitudinal symptom changes in young people with autism spectrum disorder

**DOI:** 10.1038/s41398-019-0418-5

**Published:** 2019-02-12

**Authors:** Hsiang-Yuan Lin, Alistair Perry, Luca Cocchi, James A. Roberts, Wen-Yih Isaac Tseng, Michael Breakspear, Susan Shur-Fen Gau

**Affiliations:** 1 Department of Psychiatry, National Taiwan University Hospital, and College of Medicine, Taipei, Taiwan; 2 Systems Neuroscience Group, QIMR Berghofer Medical Research Institute, Brisbane, QLD Australia; 3 Max Planck UCL Centre for Computational Psychiatry and Ageing Research, Max Planck Institute for Human Development, Berlin, Germany; 4 Center for Lifespan Psychology, Max Planck Institute for Human Development, Berlin, Germany; 5 Clinical Brain Network Team, QIMR Berghofer Medical Research Institute, Brisbane, QLD Australia; 6 Brain Modeling Team, QIMR Berghofer Medical Research Institute, Brisbane, QLD Australia; 7 Institute of Medical Device and Imaging, National Taiwan University College of Medicine, Taipei, Taiwan; 8 Graduate Institute of Brain and Mind Sciences, National Taiwan University College of Medicine, Taipei, Taiwan; 9 Metro North Mental Health Service, The Royal Brisbane and Woman’s Hospital, Brisbane, QLD Australia; 10 University of Newcastle, Newcastle, NSW Australia

## Abstract

Structural neuroimaging studies suggest altered brain maturation in autism spectrum disorder (ASD) compared with typically developing controls (TDC). However, the prognostic value of whole-brain structural connectivity analysis in ASD has not been established. Diffusion magnetic imaging data were acquired in 27 high-functioning young ASD participants (2 females) and 29 age-matched TDC (12 females; age 8–18 years) at baseline and again following 3–7 years. Whole-brain structural connectomes were reconstructed from these data and analyzed using a longitudinal statistical model. We identified distinct patterns of widespread brain connections that exhibited either significant increases or decreases in connectivity over time (*p* < 0.001). There was a significant interaction between diagnosis and time in brain development (*p* < 0.001). This was expressed by a decrease in structural connectivity within the frontoparietal network—and its broader connectivity—in ASD during adolescence and early adulthood. Conversely, these connections increased with time in TDC. Crucially, stronger baseline connectivity in this subnetwork predicted a lower symptom load at follow-up (*p* = 0.048), independent of the expression of symptoms at baseline. Our findings suggest a clinically meaningful relationship between the atypical development of frontoparietal structural connections and the dynamics of the autism phenotype through early adulthood. These results highlight a potential marker of future outcome.

## Introduction

Despite being largely diagnosed in early childhood, individuals with autism spectrum disorder (ASD) continue to experience changes in symptoms and reciprocal social interactions through adolescence to adulthood. Although these changes are clinically important, their nature and causes are not well-characterized^1,2^. The human connectome, comprising white matter tracts linking cortical and subcortical structures, also undergoes extensive reorganization and waves of maturation from late childhood, through adolescence to early adulthood^3^. Elucidating similarities and divergences between atypical and typical brain development during this critical period may enable a better understanding of ASD and enhance strategies aiming to enhance resilience and adaption^4^.

Structural neuroimaging studies, principally from cross-sectional studies, have suggested altered brain maturation in ASD compared with typically developing controls (TDC)^5,6^, characterized by early overgrowth in gray matter morphometry, followed by arrested growth throughout later childhood, and atypical decline across the remaining lifespan. Nonetheless, with very few exceptions^6^, such cross-sectional data are insufficient to infer developmental trajectories and causal relationships^4^. The development of white matter connectivity speaks to coordinated gray matter growth and functional network formation, but few studies of brain maturation in ASD have been focused here^7^. Based on limited longitudinal studies, ASD appears to be associated with the atypical development of corpus callosum microstructure^8^ and volume^9^ in early childhood, and a reduced rate of total cortical white matter growth from late childhood to adulthood^6^. Notably, the a priori selection of specific regions of interest in these studies may unnecessarily narrow the search for autism-related white matter development. An unbiased whole-brain connectomic approach would help here. However, there is little knowledge regarding longitudinal changes in whole-brain structural connectivity (the connectome) in ASD from late childhood to young adulthood. Although the study of white matter development in infants is able to predict later ASD diagnosis and severity^10,11^, the clinical significance of altered developmental trajectories of structural connectivity during late maturation in ASD remains elusive.

Here, we leveraged recent advances in diffusion-imaging and brain network analyses to report the first longitudinal investigation of structural connectome development from late childhood to early adulthood in a well-characterized high-functioning cohort of ASD and age-matched TDC. Crucially, the follow-up latency of our cohort ranged from 3 to 7 years, encompassing the most critical period bridging late childhood to young adulthood. We hypothesized that the distinct development of the white matter organization in individuals with ASD would predict the later expression of symptoms.

## Methods

### Participants and procedures

**Fig. 1:**
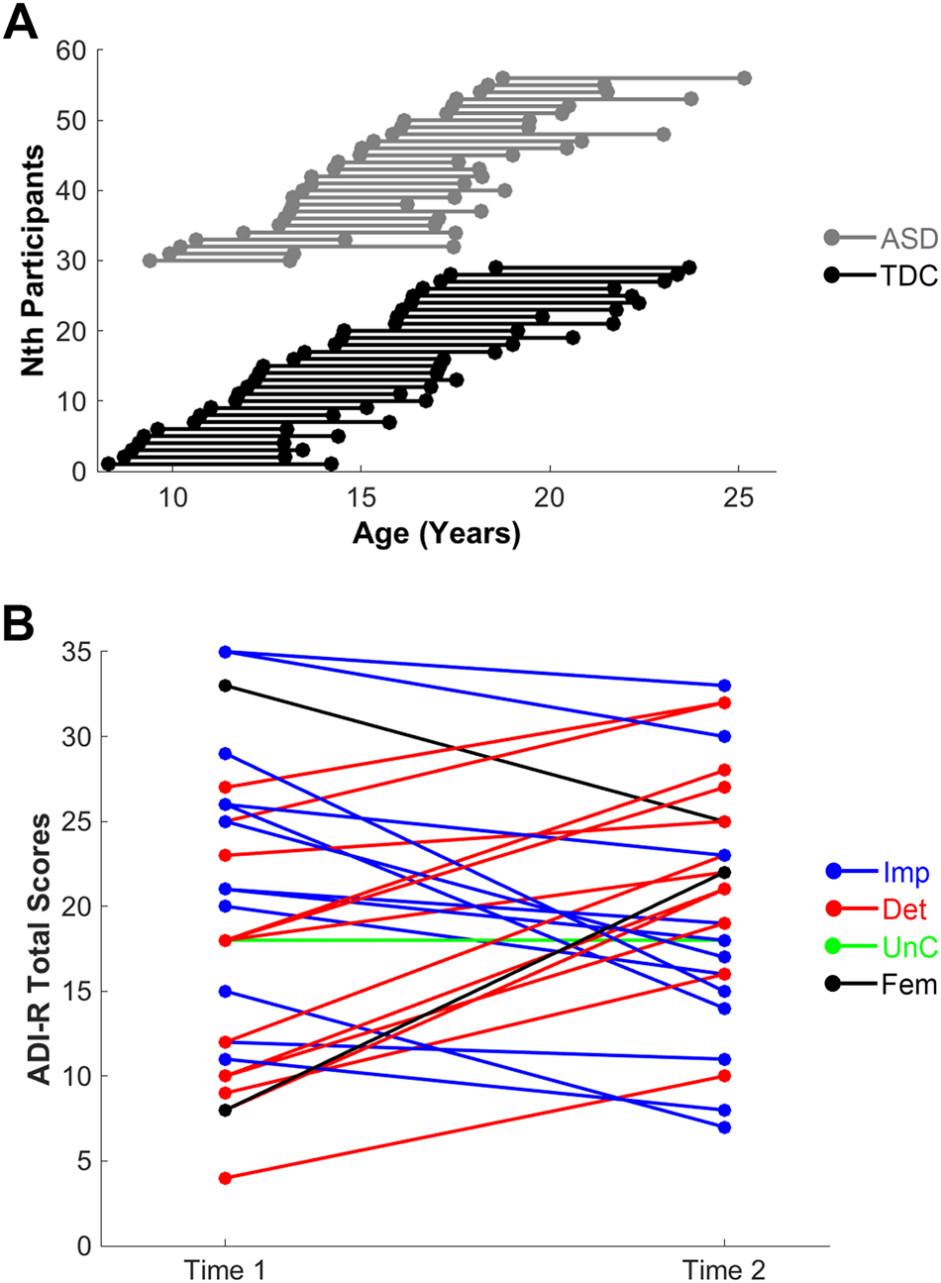
Age distributions and symptom changes among participants **a** depicts the distribution of participants’ age at assessments and follow-up latency. The black color represents the typically developing control (TDC) group; the gray color denotes the autism spectrum disorder (ASD) group. **b** depicts changes in autism symptoms, as represented by total scores on Current Behavior Algorithms of the Autism Diagnostic Interview-Revised (ADI-R) over time. For the male participants, the blue color denotes an improvement (Imp) in symptoms; the red color indicates deteriorating (Det) symptoms over time; the green color represents unchanged (Unc) autistic severity between two assessments. The black color indicates female (Fem) participants

Thirty high-functioning youths with ASD and 31 age-matched TDC (age 8–18 years, Table 1 for details) were assessed at baseline at the Department of Psychiatry, National Taiwan University Hospital, Taipei, Taiwan. Participants were recruited via advertisement at the hospital, schools, and websites. All participants were evaluated again with a follow-up latency of 3–7 years (age 13–25 years; Fig. 1a). At both time points, all participants were clinically assessed by a senior child psychiatrist to confirm the diagnosis of ASDs (Autistic disorder or Asperger’s disorder based on the DSM-IV-TR criteria) and/or other co-occurring psychiatric conditions. TDC disavowed any current or lifetime DSM-IV-TR psychiatric disorder based on clinical evaluation. Exclusion criteria for all the participants included: any prior systemic medical illness; a history of affective disorders, psychosis, substance use disorder; current depressive/anxious symptoms or suicidal ideation; current use of psychotropic medication, except methylphenidate, and a full intelligence quotient (IQ) < 70, as assessed using the Wechsler Intelligence Scale for Children–3rd Edition (age < 16years) or Wechsler Adult Intelligence Scale-3rd Edition (age ≥ 16years) at baseline.

**Table 1:** Demographic and clinical characteristics of the participants

	ASD (*N* = 27)		TDC (*N* = 29)		Statistics^a^
T1 Age (years)	14.4 (2.6); range: 9.4–18.4		13.1 (3.0); range: 8.3–18.6		*p* = 0.094
T2 Age (years)	18.8 (2.9); range: 13.1–25.2		18.0 (3.5); range: 12.9–22.7		*p* = 0.352
Follow-up latency (years)	4.4 (1.3); range: 3.0–7.2		4.9 (0.8); range: 3.4–6.1		*p* = 0.089
Sex (M/F)	25/2		17/12		*p* = 0.003
FIQ	101.3 (19.1)		111.3 (12.3)		*p* = 0.022
VIQ	102.8 (20.7)		110.3 (12.7)		*p* = 0.111
PIQ	99.7 (19.7)		110.9 (12.1)		*p* = 0.016
	T1	T2	T1	T2	
Signal dropout counts^b^	35.4 (20.1)	38.8 (23.6)	36.0 (21.4)	34.2 (22.1)	*p* = 0.899^c^
Raw connection density	37.85 (5.59)	39.99 (5.69)	38.75 (6.00)	42.57 (6.87)	*p* = 0.023^d^
Thresholded density	31.15 (3.16)	32.08 (3.10)	31.81 (3.47)	33.67 (3.82)	*p* = 0.045^e^
Autism Diagnostic Interview-Revised^f^					
Total	19.1 (8.7)	20.4 (7.2)	-	-	*p* = 0.400^g^
Social	8.3 (4.6)	10.1 (3.9)	-	-	*p* = 0.019^g^
Communication	6.3 (3.1)	7.0 (3.2)	-	-	*p* = 0.294^g^
Repetitive behavior	4.6 (3.2)	3.3 (2.3)	-	-	*p* = 0.049^g^

^a^Uncorrected for multiple tests

^b^A summary estimate of in-scanner motion levels (see the Methods)

^c^One-way ANOVA

^d^One-way ANOVA here. The linear mixed-effect model showed a significant effect of time (*p* *=* 0.001), but no effect of diagnosis (*p* = 0.118)

^e^One-way ANOVA here. The linear mixed-effect model showed a significant effect of time (*p* *=* 0.007), but no effect of diagnosis (*p* = 0.086)

^f^Current behavior algorithms

^g^Pairwise *t* test

Parents of youths with ASD received the Chinese version of the Autism Diagnostic Interview-Revised (ADI-R)^12^ to further confirm the ASD diagnosis at baseline, and again at follow-up to assess current symptoms using the Current Behavior Algorithms. At both baseline and follow-up, parents of all participants also received the Chinese version of the Schedule for Affective Disorders and Schizophrenia for School-Age Children–Epidemiological Version interview to further provide clinical diagnostic evaluations^13^.

Of the participants with ASD, eight had co-occurring attention-deficit hyperactivity disorder (ADHD; six of them still had a full diagnosis at follow-up, whereas the other two youths had partial remission from ADHD diagnosis). Three participants with co-occurring ASD and ADHD had taken methylphenidate. Another two youths with ASD had the co-occurring oppositional defiant disorder at baseline, but this comorbidity remitted at follow-up. Another three youths with ASD had a history of adjustment disorder between the follow-up periods.

All study procedures were approved by the Research Ethics Committee of the National Taiwan University Hospital, Taipei, Taiwan (Approval number, 201403109RINC; ClinicalTrials.gov number, NCT02233348). All participants and their parents provided written informed consent.

### Imaging data acquisition

Diffusion spectrum imaging (DSI; pulsed-gradient spin-echo diffusion echo planar imaging sequence with a twice-refocused balanced echo; repetition time/echo time = 9600/130 ms, slice thickness = 2.5 mm, acquisition matrix = 80 × 80, field of view = 200 × 200 mm, in-plane spatial resolution = 2.5 mm × 2.5 mm, 101 diffusion-encoding directions covering a half q-space 3D grid^14^ with radial grid size of 3, b_max_ = 4000 s/mm^2^) and T1-weighted images (magnetization-prepared rapid gradient echo sequence; repetition/echo time = 2000/2.98 msec; flip angle = 9°; matrix size = 256 × 256; inversion time = 900 msec; voxel size = 1 mm^3^) were longitudinally acquired in the same Siemens 3 T Tim Trio scanner using a 32-channel head coil at the Advanced Biomedical Imaging Lab of the NTUH.

### DSI data processing and connectome construction

The DSI data first underwent a quality assurance procedure to ensure acceptable in-scanner head motion: Each DSI image of the individual (54 slices × (101 directions DW images + 1 null image) = 5508 images) was scrutinized by calculating signals in the central square (20 × 20 pixels) of each image. Signal loss was defined as the average signal intensity of an image lower than two standard deviations from the mean of all images (after correcting for its b value)^15^. As jerky head motion induces signal loss in DSI images, these signal dropout counts were considered a proxy estimate for overall levels of in-scanner head motions. Individuals of DSI data with > 90 images of signal loss, at either baseline or follow-up, were excluded from further analyses^15^, resulting in a final sample of 27 youths with ASD (25 males) and 29 TDC (17 males) (Table 1).

DSI data were reconstructed using the *q*-space diffeomorphic reconstruction (QSDR) approach implemented in DSI Studio (www.dsi-studio.labsolver.org)^16^. QSDR first computed the quantitative anisotropy in each voxel in native space. Then the reconstructed images were warped to a template quantitative anisotropy volume in Montreal Neurological Institute (MNI) space using a nonlinear registration algorithm implemented in Statistical Parametric Mapping. In MNI space, a diffusion sampling length ratio of 1.25 mm with five fiber orientation per voxel and eightfold orientation distribution function tessellation was used to obtain the spin distribution function, and the output resolution was 2 mm. A deterministic fiber tracking algorithm^17^ was performed with extreme turning angle threshold of 60°, step size of 1.0 mm, minimum and maximum length of 10 and 400 mm, respectively. In total, 10,000,000 streamlines were seeded throughout the whole-brain and terminated when the local quantitative anisotropy fell below values estimated using Otsu’s threshold^17^, which gives the optimal separation between background and foreground. Other tracking parameters as specified in DSI Studio were: Smoothing: 0; Seed orientation: all; Seed position: subvoxel; Randomize seeding: off; Direction interpolation: trilinear.

To generate inter-areal structural connectivity estimates, a cortical atlas defined by clusters of homogenous intrinsic functional connectivity signals^18^ was overlaid with subcortical and basal-ganglia regions (https://fsl.fmrib.ox.ac.uk/fsl/fslwiki/Atlases) (Supplementary Table 1). These pre-defined anatomical boundaries were combined with the individual’s whole-brain tractography maps to generate a weighted structural network (a 114 × 114 weighted structural connectivity matrix). Each network edge corresponds to the total number of normalized streamlines that interconnect two regions, adjusted for the interregional fiber length. The connection density of these matrices ranged from 28 to 55%. To balance false positives and false negatives of connectome reconstruction^19^, preserve within-group intersubject variability and preserve weights of long-range connections^20^, these structural networks were thresholded using a consistency-based thresholding approach^20^, based upon the 50% most-consistent connections for the respective group and study interval. The connection density of these thresholded matrices ranged from 25 to 40%. Connection matrices at follow-up were denser than those at baseline (Table 1).

### Network-based statistics

Network-based statistics (NBS)^21^ were employed to identify subnetworks of brain connections that exhibit significant main effects of time (i.e., baseline compared with follow-up), diagnosis, and their interactions. NBS is a nonparametric method based on the principles underpinning traditional cluster-based thresholding of statistical parametric maps and hence proceeds to identify subnetworks of topologically connected suprathreshold connections. Networks were permuted 10,000 times to obtain an empirical null distribution in order to control for family-wise error (FWE) over a large number of connectome edges. We computed a repeated-measures analysis of variance for the effects of time, diagnosis, and diagnosis by time interaction. We applied a threshold of an FWE-corrected *p* < 0.05 to the resulting *t* statistic matrix (based on a height threshold of *t* = 3.0, the default optimization within NBS, corresponding to uncorrected *p* = 0.002) to yield a binarized matrix of suprathreshold connections. Results corresponding to different height thresholds are provided in Supplementary Figs. 3 and 5 (*t* = 2.8) and 4 and 6 (*t* = 3.2), respectively. Nuisance regressors comprised sex, in-scanner motion (signal dropout counts) and follow-up latency.

### Associations between longitudinal measures of brain and symptoms

To assess associations between longitudinal connectivity measures and overall ASD symptoms, we used the cross-lagged panel model^22^, a variant of structural equation modeling (SEM). This model allows concurrent tests for associations between repeatedly measured variables. The first cross-lagged coefficient represents the association between connectivity weights estimated at baseline and ASD severity estimated at follow-up that had been adjusted for ASD severity measured at baseline. The other cross-lagged coefficient represents the association between overall levels of autism symptoms measured at baseline and connectivity weights measured at follow-up, adjusted for baseline connectivity weights. Cross-sectional correlations between connectivity weights and ASD symptoms were also modeled (only the association at baseline was reported, as the association at follow-up simply indicates the correlation in the residual terms). Finally, autoregressive coefficients representing the changes of autism symptoms and connectivity weights from baseline to follow-up, respectively, were modeled. This SEM model was also adjusted for nuisance covariates as used in the NBS. Test statistics were estimated using bootstrapping methods (5000 bootstrap samples). The cross-lagged panel model used here was within the R package “lavaan” (http://lavaan.ugent.be/),

## Results

### Demographic and clinical presentations

There were no significant differences between the ASD and TDC groups for age at baseline or follow-up, follow-up latency, or signal dropout counts. The TDC group demonstrated greater full-scale IQ, performance IQ, and a greater proportion of females than the ASD group (Table 1).

All individuals with ASD at baseline met DSM-5 ASD diagnostic criteria at follow-up^23^. As estimated by the Current Behavior Algorithms of the ADI-R, 48% of participants (one female in this subgroup) showed improvements at follow-up, 48% exhibited more severe symptoms (the other female in this subgroup), whereas 4% remained unchanged (Fig. 1b and Supplementary Table 2). Furthermore, the ASD group demonstrated a decline in social interaction (*p* = 0.019), improvements in repetitive/stereotyped behaviors and interests (*p* = 0.049) but no changes in communication (*p* = 0.294) (Table 1; see also Supplementary Table 3 and Supplementary Fig. 1).

### Diagnosis, time, and diagnosis by time effects on the connectome

**Fig. 2:**
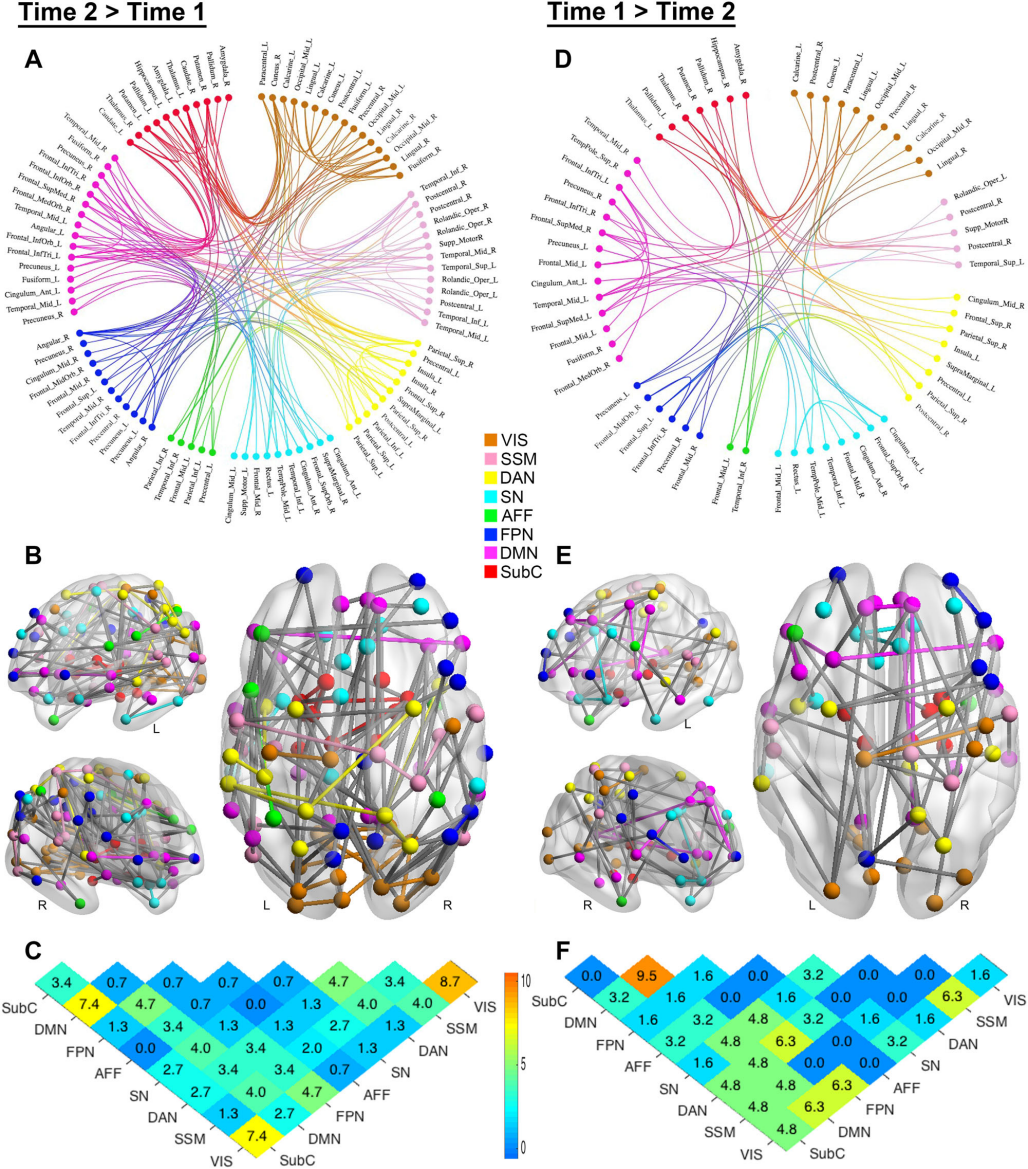
Developmental changes in structural connectome (main effect of time) **a**, **b**, **d**, **e** Anatomical arrangement of nodes and edges identified by NBS exhibiting increases (**a**, **b**; 149 edges linking 94 nodes) and decreases (**d**, **e** 63 edges linking 61 nodes) in normalized streamlines (adjusted for the interregional fiber length) over time. Regions of interest in connectograms are grouped per functional divisions^18^, with the node color denoting the functional assignment of each region. Anatomical assignments are based on the Automated Anatomical Labeling atlas (http://www.gin.cnrs.fr/en/tools/aal-aal2/). The location of the node in **b** and **e** represents the MNI centroid coordinates of each parcellated region. **c**, **f** Distribution of edges based on the functional divisions they interconnect. Values in the matrices represent relative proportions (percentage), calculated as the ratio between the frequency of edges linking each pair of divisions, and the total number of edges exhibiting increases and decreases over time, respectively. L = left; R = right; Mid = middle; Inf = inferior; Tri = pars triangularis; Orb = orbital; Sup = superior; Oper = operculum; VIS = visual network; SSM = sensory-somatomotor network; DAN = dorsal attention network; SN = salience/ventral attention network; AFF = affective network; FPN = frontoparietal network; DMN = default-mode network; SubC = subcortical

The contrast for the main effect of time revealed two distributed networks, with widespread increases in structural connectivity between baseline and follow-up in one network (FWE-corrected *p* < 0.001; Fig. 2a–c) and decreases in the other (FWE-corrected *p* < 0.001; Fig. 2d–f). These encompassed most major functional systems of the brain. These changes of connectivity over time were comparable across the diagnosis groups (Supplementary Fig. 2), reflecting the substantial neurodevelopmental changes across this critical age bracket. There was no significant effect of ASD diagnosis (FWE-corrected *p* = 0.899).

**Fig. 3:**
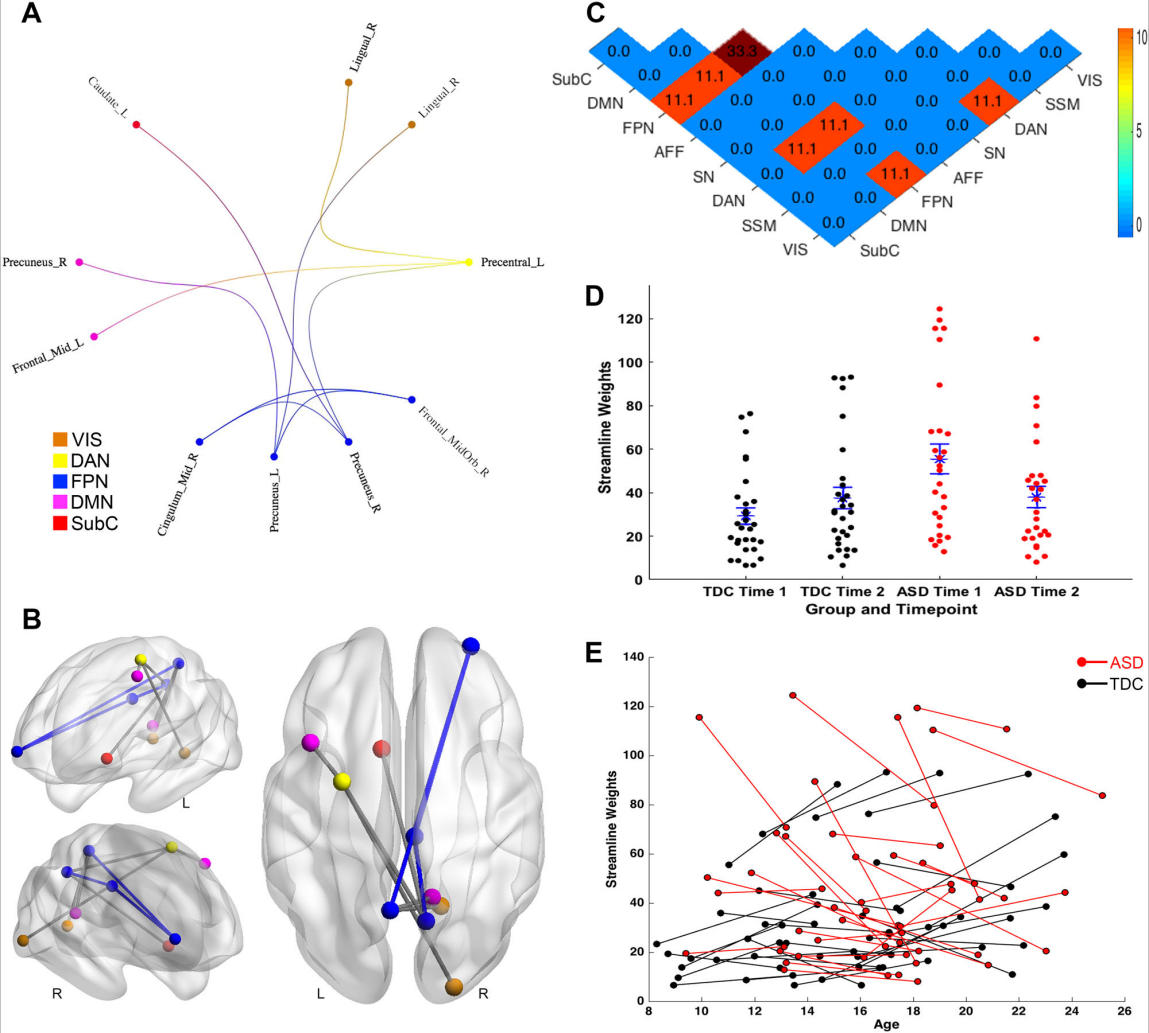
Distinct developmental changes in structural connectome of autism spectrum disorder (time by diagnosis interaction) Similar to Fig. 1, **a***–***c** illustrate the anatomical arrangement of 10 nodes and 9 edges of the subnetwork identified by NBS as exhibiting a significant diagnosis by time interaction in normalized streamlines. **d**–**e** Distribution of streamline weights of this subnetwork exhibiting a diagnosis by time interaction, and changes of these streamline weights over time. **d** blue asterisks denote mean, where blue bars correspond to standard error of group-wise streamline weights. ASD = autism spectrum disorder; TDC = typically developing control; L = left; R = right; Mid = middle; Orb = orbital; VIS = visual network; DAN = dorsal attention network; FPN = frontoparietal network; DMN = default-mode network; SubC = subcortical.; TDC = typically developing control

The group by time interaction revealed a statistically significant subnetwork comprising connections within the frontoparietal network and between this network and other brain networks including visual, dorsal attention, default-mode networks, and subcortical regions (10 nodes and 9 edges; FWE-corrected *p* < 0.001; Fig. 3a–c). This interaction was associated with a decrease in structural connectivity in ASD during adolescence and early adulthood among connections that otherwise increased (Fig. 3d and e). Specifically, youths with ASD exhibited markedly stronger connectivity of this subnetwork at baseline, relative to neurotypical youths, then a reduction in structural connectivity at follow-up; whereas TDCs demonstrated increases in connectivity of this subnetwork over time.

To test the stability of these results, we undertook confirmatory analyses using different search thresholds (*t* values as 2.8 and 3.2) for the NBS. As shown in Supplementary Figs. 2–4, the confirmatory NBSs identified similar patterns of an increased subnetwork and decreased one in connectomic development over time. NBS results derived from a more liberal (*t* = 2.8; Supplementary Fig. 5), and a more stringent test threshold (*t* = 3.2; Supplementary Fig. 6) yielded similar subnetworks, which exhibited time by diagnosis effects and remained centered on the frontoparietal network. Consistent with the effect of increasing the network forming threshold, these subnetworks become sparser at the higher search threshold.

### Associations of changes of the connectome and autism symptoms

**Fig. 4:**
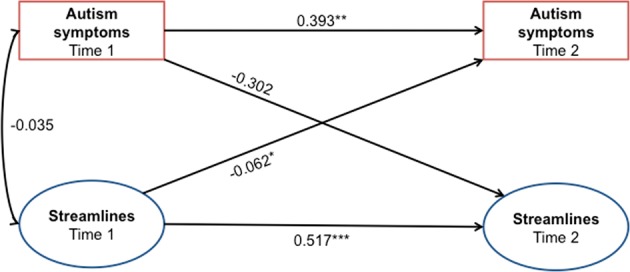
The cross-lagged panel model assessing associations between longitudinal connectivity and the ASD and phenotype Baseline streamline weights of the subnetwork exhibiting a diagnosis by time interaction was associated with overall autistic severity at follow-up. Numeric values correspond to standardized structural regression coefficients. ^*^p < 0.05; ^**^p < 0.005; ^***^p < 0.001

The cross-lagged panel model showed that ASD symptoms at baseline were positively associated with those estimated at follow-up (*p* = 0.003). Similarly, baseline connectivity weights of this subnetwork were also positively associated with follow-up connectivity (*p* < 0.001). However, notably, baseline connectivity strength (in the subnetwork showing a diagnosis by time effect; Fig. 3a–c) was negatively associated with follow-up phenotypic ASD features, after adjusting for baseline symptom levels, in-scanner motion, sex, and follow-up latency (*p* = 0.048; Fig. 4): That is, stronger expression of this network at baseline in the ASD participants predicted a lesser symptom load at follow-up, independent of the expression of symptoms at baseline. Levels of overall ASD symptoms at baseline were not associated with streamline weights of this subnetwork at baseline cross-sectionally, nor with connections at follow-up. The overall cross-lagged panel model showed a good fit to the data (see Supplementary Table 4 for fit indices)^24^.

### Testing the effects of sex and comorbidity on diagnosis by time interaction

We implemented a post-hoc analysis of the sex effect on the diagnosis by time interaction by computing sex-stratified pairwise *t*-tests of connectivity strength in the subnetwork showing a diagnosis by time effect (Fig. 3a–c). As shown in Supplementary Table 5, across the ASD and TDC groups, the Chi-square statistics for this test were not statistically significant (*p* = 0.326 and *p* = 0.913, respectively). This indeed suggests that the diagnosis by time interaction was present in both sexes. An auxiliary NBS, including an additional categorical nuisance covariate denoting the status of co-occurring psychiatric conditions and psychostimulant treatment, was also implemented to address potential comorbidity effects. This analysis yielded a subnetwork exhibiting a significant time by diagnosis interaction, with a similar spatial extent that primarily involved the frontoparietal system (*p* = 0.039, FWE-corrected, Supplementary Fig. 7). The cross-lagged panel model based on this sparser subnetwork still showed that baseline connectivity strength was negatively associated with follow-up autistic levels. These auxiliary results again support the conclusion that the main findings arise from effects of autism per se.

## Discussion

This longitudinal assessment of whole-brain structural connectivity in ASD highlights two key findings: (i) ASD impacts the development of within- and between-connectivity of the frontoparietal system in adolescence and early adulthood. Specifically, we observed hyperconnectivity of this subnetwork at baseline in the ASD group, followed by an adaptive pruning over time; (ii) these aberrant frontoparietal hyperconnections at baseline predict relatively better outcome of the autism phenotype at follow-up, suggesting that an adaptive pruning process in this subnetwork between late childhood to early adulthood confers improvements in symptoms.

The frontoparietal network is involved in regulating a diversity of cognitive processes, harnessing adaptive interactions between different brain systems according to contextual demands^25^. Altered frontoparietal network activity has been observed in ASD, with weaker cognitive control associated with poorer clinical outcomes^25^. Our longitudinal results highlight the central role of frontoparietal structural connectivity, along with its links to other major brain networks, on the ASD phenotype across a crucial developmental phase, from late childhood through to early adulthood. The fact that stronger frontoparietal connections in ASD at baseline predicted lower follow-up symptom load, along with the background higher baseline connectivity (Fig. 2d and e), suggests that remodeling of this network may represent an adaptive pathway following early atypical neural processes^26^. Moreover, consistent with prior work^10,27^, this result highlights the potential of leveraging brain-based statistical models to improve the predictive power of ASD outcomes, beyond cognitive, and demographic features^1,2^.

We identified marked increases and decreases in structural connectivity development between baseline and follow-up periods across both groups (Supplementary Fig. 2). These results, largely consistent with the earlier empirical evidence^7,28^, highlight that adolescence is characterized by selective maturation changes of structural connections between functionally distributed regions. Specifically, increases in connectivity with development involved extensive connections between- and within-major brain networks. The subnetwork showing decreases in connectivity development was mostly noted in between-system connections, with fewer edges compared with those in the subnetwork showing increase strengths across time. The reorganization of connectome across this critical period was not restricted to any particular functional system. Across both time samples, the ASD group did not exhibit significant differences in the connectome compared with TDC, which is inconsistent with the earlier findings of diffuse white matter disruption^29^. Given the careful quality control for DSI data and tight motion-matching in the current data, as well as comparable sample sizes and high-functioning cohorts with earlier studies^29^, this finding might suggest that motion confounds have inflated previously reported white matter differences in ASD at the group level^30^. The present null finding in a diagnostic effect may also highlight the advantage of performing a longitudinal analysis to capture variance across the diagnostic groups with regards to differences in their developmental trajectories. Although our analysis places probabilistic upper bounds on any putative underlying effect, a larger sample would be required to better estimate the presence and size of any subtle ASD effect.

Our longitudinal investigation lends weight to the hypothesis that ASD reflects a cascade of transient neurodevelopmental insults during a sensitive period (i.e., early in postnatal years), subsequently followed by adaptive processes^26^. Specifically, at a macroscopic level, distinct white matter development of ASD is noted as early as 6 months of age^11^. These complex remodeling of subcortical–cortical connections from late childhood to early adulthood are superimposed on broader, neurodevelopment changes shared across ASD and controls. Future longitudinal studies of white matter development in ASD from early- and mid-childhood through to early adulthood would better disambiguate the complete trajectory of this complex process of insult and adaptive response^26^.

The parent-reported longitudinal assessments revealed that total ASD and communication symptoms did not change at follow-up, whereas impairments in social interaction increased, and those within the restricted/repetitive domain decreased. These results contrast with the majority of clinical outcome studies^1,2^, which suggest that the majority of youths with ASD demonstrate symptom improvements, especially in the communication domain, whereas remaining relatively stable in repetitive/restricted behavior domain. The selection of a cohort of ASD without intellectual disabilities might partially contribute to the disagreement with previous clinical outcome reports, as initially less-severe symptoms in some subjects in the present study might have a “ceiling effect” on symptom improvement. It is also possible that some participants might show nonmonotonic functions of improvement or decline over time, alongside the recurrent change in impairment as reported in previous studies^1,2^. However, the relatively long follow-up latency (3–7 years) and only two data-collection time points in the present study would preclude capturing these dynamic trajectories of symptom changes. Future richly phenotyped investigations with regular follow-up intervals are recommended to disambiguate these possibilities. Nonetheless, there is considerable heterogeneity in every behavioral domain associated with impairments in ASD. Etiologies driving these different patterns in the development of phenotypic expressions await further investigation.

Several other methodological limitations are noted. First, although our sample size is comparable to most other brain imaging longitudinal studies of ASD, it is nonetheless relatively moderate^1,11,27^. Clearly larger cohorts are more sensitive to weaker effects that we may have missed and are also able to provide more accurate estimates of the size of those stronger effects present in studies of modest size^31^. Larger studies are also able to disentangle sex, psychiatric comorbidity, and medication effects, although they typically lack the tight quality control achievable in a single-site sample like ours. Smaller studies are able to identify large, and clinically meaningful effects and influence the design and hypothesis-driven interrogation of larger, consortia studies. For example, a functional connectivity “fingerprints” of attention trained on a relatively small healthy cohort (*N* = 25) was able to predict individual variation in attention in a much larger clinical (ADHD) cohort^32^. These are important considerations, given the absence of currently available longitudinal diffusion data in world-wide multi-site databases, i.e., Autism Brain Imaging Data Exchange I and II^33^, as well as the Enhancing Neuroimaging Genetics Through Meta-Analysis, ENIGMA, ASD working group^34^. Second, there was an uneven sex ratio in the ASD group. Although our auxiliary analyses yielded that both sexes showed similar patterns in the diagnosis by time effect, more nuanced sex differences in brain development in ASD do likely exist^35,36^ and should be explored in the further studies designed for this specific purpose. Third, the longitudinal assessment did not occur at a fixed time interval but over a broader range. At last, the study lacked assessments of hormonal and other effects of puberty, which should be included in the future studies to address the related effects.

As the first longitudinal study of connectomic changes in young people with ASD, our findings suggest a novel relationship between structural connectivity and the dynamics of the autism phenotype through early adulthood. These analyses persisted when accounting for potential confounding effects in our heterogeneous sample. Future multi-disorder studies are required to more specifically disambiguate these issues. In particular, our findings highlight a prominent role of structural connectivity within the frontoparietal network—and its broader connectivity—in shaping the phenotypic development into late adolescence and early adulthood. Our finding marks an important benchmark in the pursuit of imaging biomarkers of ASD resilience and adaption.

## Supplementary information

Supplementary Information
