# Supplementary material for: Development of frontoparietal connectivity predicts longitudinal symptom changes in young people with autism spectrum disorder
Source: Transl Psychiatry. 2019 Feb 12;9:86. doi: 10.1038/s41398-019-0418-5 (PMC6372645; doi:10.1038/s41398-019-0418-5)
Supplement: Supplementary file 1 — Supplementary Information [file 41398_2019_418_MOESM1_ESM.docx]

**Supplementary Information**

**Distinct development of frontoparietal structural connectivity predicts longitudinal symptom changes in youths with autism spectrum disorder**

Hsiang-Yuan Lin, MD^1,2^, Alistair Perry, PhD^2,3,4^, Luca Cocchi, PhD^5^, James Roberts, PhD^6^, Wen-Yih Isaac Tseng, MD, PhD^7,8^, Michael Breakspear, MBBS, PhD, FRANZCP^2,9*^, Susan Shur-Fen Gau, MD, PhD^1,8*^

**Supplementary Table**

**Supplementary Table 1.** *Center of mass and corresponding AAL match for each parcellation region.*

| **#** | **X** | **Y** | **Z** | **AAL Label** | **Network** | **#** | **X** | **Y** | **Z** | **AAL Label** | **Network** |
| --- | --- | --- | --- | --- | --- | --- | --- | --- | --- | --- | --- |
| 1 | -25 | -77 | -14 | Fusiform_L_1 | VIS | 58 | 41 | -22 | 60 | Postcentral_R_1 | VIS |
| 2 | -27 | -94 | -5 | Occipital_Mid_L_1 | VIS | 59 | 30 | -37 | 64 | Postcentral_R_2 | SSM |
| 3 | -5 | -92 | -3 | Calcarine_L_1 | VIS | 60 | 11 | -24 | 65 | Supp_Motor_Area_R_1 | SSM |
| 4 | -26 | -88 | 20 | Occipital_Mid_L_2 | VIS | 61 | 53 | -16 | 6 | Temporal_Sup_R_1 | SSM |
| 5 | -16 | -61 | -7 | Lingual_L_1 | VIS | 62 | 41 | -15 | 15 | Rolandic_Oper_R_1 | SSM |
| 6 | -12 | -65 | 6 | Calcarine_L_2 | VIS | 63 | 57 | -4 | 12 | Rolandic_Oper_R_2 | SSM |
| 7 | -7 | -81 | 26 | Cuneus_L_1 | VIS | 64 | 57 | -5 | 31 | Postcentral_R_3 | SSM |
| 8 | -39 | -23 | 59 | Postcentral_L_1 | VIS | 65 | 49 | -60 | -11 | Temporal_Inf_R_1 | SSM |
| 9 | -11 | -26 | 64 | Paracentral_Lobule_L_1 | VIS | 66 | 51 | -63 | 16 | Temporal_Mid_R_1 | SSM |
| 10 | -53 | -22 | 8 | Temporal_Sup_L_1 | SSM | 67 | 27 | -67 | 50 | Parietal_Sup_R_1 | DAN |
| 11 | -37 | -21 | 16 | Rolandic_Oper_L_1 | SSM | 68 | 50 | -24 | 42 | Postcentral_R_4 | DAN |
| 12 | -54 | -11 | 14 | Rolandic_Oper_L_2 | SSM | 69 | 15 | -53 | 66 | Parietal_Sup_R_2 | DAN |
| 13 | -55 | -8 | 33 | Postcentral_L_2 | SSM | 70 | 28 | -2 | 59 | Frontal_Sup_R_1 | DAN |
| 14 | -47 | -57 | -13 | Temporal_Inf_L_1 | SSM | 71 | 60 | -26 | 28 | SupraMarginal_R_1 | DAN |
| 15 | -47 | -71 | 11 | Temporal_Mid_L_1 | SSM | 72 | 40 | 8 | 1 | Insula_R_1 | DAN |
| 16 | -25 | -68 | 48 | Parietal_Sup_L_1 | DAN | 73 | 11 | -30 | 45 | Cingulum_Mid_R_1 | DAN |
| 17 | -57 | -25 | 39 | Parietal_Inf_L_1 | DAN | 74 | 8 | 6 | 53 | Supp_Motor_Area_R_2 | SN |
| 18 | -42 | -34 | 48 | Postcentral_L_3 | DAN | 75 | 58 | -39 | 43 | SupraMarginal_R_2 | SN |
| 19 | -22 | -50 | 66 | Parietal_Sup_L_2 | DAN | 76 | 32 | 46 | 29 | Frontal_Mid_R_1 | SN |
| 20 | -27 | -3 | 59 | Precentral_L_1 | DAN | 77 | 6 | 27 | 32 | Cingulum_Ant_R_1 | SN |
| 21 | -59 | -38 | 30 | SupraMarginal_L_1 | DAN | 78 | 13 | 37 | -19 | Frontal_Sup_Orb_R_1 | SN |
| 22 | -42 | -2 | -7 | Insula_L_1 | DAN | 79 | 38 | 0 | -35 | Temporal_Inf_R_2 | AFF |
| 23 | -38 | 12 | 6 | Insula_L_2 | DAN | 80 | 39 | -45 | 49 | Parietal_Inf_R_1 | AFF |
| 24 | -11 | -34 | 46 | Cingulum_Mid_L_1 | SN | 81 | 45 | 39 | 15 | Frontal_Inf_Tri_R_1 | FPN |
| 25 | -6 | 3 | 61 | Supp_Motor_Area_L_1 | SN | 82 | 49 | 10 | 27 | Precentral_R_2 | FPN |
| 26 | -30 | 44 | 30 | Frontal_Mid_L_1 | SN | 83 | 61 | -23 | -18 | Temporal_Mid_R_2 | FPN |
| 27 | -5 | 20 | 34 | Cingulum_Ant_L_1 | SN | 84 | 45 | -62 | 46 | Angular_R_1 | FPN |
| 28 | -14 | 32 | -20 | Rectus_L_1 | SN | 85 | 43 | 17 | 45 | Frontal_Mid_R_2 | FPN |
| 29 | -33 | 1 | -35 | Temporal_Pole_Mid_L_1 | SN | 86 | 30 | 57 | -3 | Frontal_Mid_Orb_R_1 | FPN |
| 30 | -57 | -32 | -21 | Temporal_Inf_L_2 | SN | 87 | 5 | -27 | 33 | Cingulum_Mid_R_2 | FPN |
| 31 | -37 | -54 | 46 | Parietal_Inf_L_2 | AFF | 88 | 10 | -65 | 42 | Precuneus_R_1 | FPN |
| 32 | -43 | 33 | 20 | Frontal_Mid_L_2 | AFF | 89 | 55 | -51 | 31 | Angular_R_2 | FPN |
| 33 | -48 | 6 | 28 | Precentral_L_2 | AFF | 90 | 26 | 24 | 49 | Frontal_Mid_R_3 | DMN |
| 34 | -23 | 60 | -1 | Frontal_Sup_L_1 | FPN | 91 | 7 | -53 | 30 | Precuneus_R_2 | DMN |
| 35 | -10 | -73 | 37 | Precuneus_L_1 | FPN | 92 | 7 | 48 | 1 | Frontal_Med_Orb_R_1 | DMN |
| 36 | -6 | -60 | 56 | Precuneus_L_2 | FPN | 93 | 12 | 46 | 40 | Frontal_Sup_Medial_R_1 | DMN |
| 37 | -4 | -26 | 33 | Cingulum_Mid_L_2 | FPN | 94 | 35 | 27 | -14 | Frontal_Inf_Orb_R_1 | DMN |
| 38 | -25 | 20 | 51 | Frontal_Mid_L_3 | DMN | 95 | 51 | 28 | 0 | Frontal_Inf_Tri_R_2 | DMN |
| 39 | -6 | -53 | 33 | Precuneus_L_3 | DMN | 96 | 13 | -54 | 15 | Precuneus_R_3 | DMN |
| 40 | -6 | 47 | -1 | Cingulum_Ant_L_2 | DMN | 97 | 32 | -31 | -21 | Fusiform_R_2 | DMN |
| 41 | -55 | -3 | -20 | Temporal_Mid_L_2 | DMN | 98 | 51 | 6 | -17 | Temporal_Pole_Sup_R_1 | DMN |
| 42 | -58 | -32 | -2 | Temporal_Mid_L_3 | DMN | 99 | 57 | -26 | -2 | Temporal_Mid_R_3 | DMN |
| 43 | -47 | -63 | 36 | Angular_L_1 | DMN | 100 | 58 | -42 | 13 | Temporal_Mid_R_4 | DMN |
| 44 | -10 | 45 | 40 | Frontal_Sup_Medial_L_1 | DMN | 101 | -10 | -19 | 7 | Thalamus_L_1 | SubC |
| 45 | -41 | 14 | 48 | Frontal_Mid_L_4 | DMN | 102 | -13 | 10 | 10 | Caudate_L_1 | SubC |
| 46 | -35 | 22 | -11 | Frontal_Inf_Orb_L_1 | DMN | 103 | -25 | 1 | 0 | Putamen_L_1 | SubC |
| 47 | -46 | 33 | -2 | Frontal_Inf_Tri_L_1 | DMN | 104 | -19 | -5 | -1 | Pallidum_L_1 | SubC |
| 48 | -11 | -56 | 13 | Precuneus_L_4 | DMN | 105 | -26 | -22 | -15 | Hippocampus_L_1 | SubC |
| 49 | -26 | -33 | -17 | Fusiform_L_2 | DMN | 106 | -23 | -5 | -18 | Amygdala_L_1 | SubC |
| 50 | -57 | -50 | 12 | Temporal_Mid_L_4 | DMN | 107 | -10 | 12 | -7 | Caudate_L_2 | SubC |
| 51 | 27 | -65 | -12 | Fusiform_R_1 | VIS | 108 | 11 | -18 | 7 | Thalamus_R_1 | SubC |
| 52 | 22 | -94 | -4 | Lingual_R_1 | VIS | 109 | 13 | 11 | 10 | Caudate_R_1 | SubC |
| 53 | 36 | -82 | 17 | Occipital_Mid_R_1 | VIS | 110 | 26 | 2 | 0 | Putamen_R_1 | SubC |
| 54 | 8 | -76 | 5 | Calcarine_R_1 | VIS | 111 | 20 | -4 | -1 | Pallidum_R_1 | SubC |
| 55 | 17 | -58 | 6 | Lingual_R_2 | VIS | 112 | 27 | -20 | -15 | Hippocampus_R_1 | SubC |
| 56 | 13 | -86 | 29 | Cuneus_R_1 | VIS | 113 | 23 | -3 | -18 | Amygdala_R_1 | SubC |
| 57 | 46 | -11 | 48 | Precentral_R_1 | VIS | 114 | 9 | 12 | -7 | Caudate_R_2 | SubC |

VIS=visual; SSM=somatosensorimotor; DAN=dorsal attention network; SN=salience network; AFF=affective; FPN=frontoparietal network; DMN=default-mode network; SubC=subcortical; L=left; R=right; Ant=anterior; Mid=middle; Sup=superior; Inf=inferior; Orb=orbital; Tri=triangularis; Oper=operculum.

**Supplementary Table 2.** *Ratio of symptom change patterns in autism spectrum disorder.*

| % (N) | **Improvement** | **Deterioration** | **No change** |
| --- | --- | --- | --- |
| **Total** | 48 (13) | 48 (13) | 4 (1) |
| **Social Interaction** | 30 (8) | 59 (16) | 11 (3) |
| **Communication** | 33 (9) | 45 (12) | 22 (6) |
| **Repetitive/stereotyped behaviors and interests** | 56 (15) | 26 (7) | 18 (5) |

**Supplementary Table 3.** *Demographic and clinical features among subgroups of autism spectrum disorder.*

|  | **Improvement**^a^ **(N=13)** | | **Deterioration (N=13)** | | **No change (N=1)** | | **Statistics**^b^ |
| --- | --- | --- | --- | --- | --- | --- | --- |
| **T1 Age (years)** | 14.3 (2.8); range: 9.9-18.4 | | 14.5 (2.7); range: 9.4-18.2 | | 13.5 | | *p*=0.887 |
| **T2 Age (years)** | 18.7 (3.4); range: 13.2-25.2 | | 18.8 (2.5); range: 13.1-23.7 | | 18.8 | | *p*=0.960 |
| **Follow-up latency (years)** | 4.4 (1.3); range: 3.0-7.2 | | 4.3 (1.3); range: 3.4-6.1 | | 5.4 | | *p*=0.857 |
| **Sex (M/F)** | 12/1 | | 12/1 | | 1/0 | | *p*=1 |
| **FIQ** | 96.9 (18.0) | | 104.2 (20.3) | | 120 | | *p*=0.341 |
| **VIQ** | 97.7 (19.4) | | 107.6 (22.2) | | 107 | | *p*=0.237 |
| **PIQ** | 96.4 (18.7) | | 100.8 (20.1) | | 130 | | *p*=0.570 |
| **Signal dropout counts**^c^ | T1 | T2 | T1 | T2 | T1 | T2 |  |
|  | 35.3 (19.9) | 36.4 (22.7) | 35.8 (21.9) | 41.5 (26.7) | 31 | 28 | T1: *p*=0.948; T2: *p*=0.600 |
| **Autism Diagnostic Interview-Revised**^d^ | | | | | | | |
| **Total** | 23.8 (8.1) | 18.2 (7.9) | 14.6 (7.3) | 22.9 (6.1) | 18 | 18 | T1: *p*=0.006; T2: *p*=0.098 |
| **Social** | 9.9 (5.1) | 9.2 (4.6) | 6.7 (3.8) | 11.2 (2.9) | 7 | 8 | T1: *p*=0.079; T2: *p*=0.219 |
| **Communication** | 7.7 (3.3) | 5.9 (2.4) | 4.9 (2.4) | 8.2 (3.7) | 7 | 7 | T1: *p*=0.021; T2: *p*=0.080 |
| **Repetitive behavior** | 6.2 (2.9) | 3 (2.4) | 3 (2.9) | 3.6 (2.3) | 4 | 3 | T1: *p*=0.010; T2: *p*=0.515 |

^a^Subgrouping based on patterns of changes in total scores of the Autism Diagnostic Interview-Revised.

^b^Comparisons only between ‘Improvement’ and ‘Worsening’ subgroups.

^c^A summary estimate of in-scanner motion levels (see the Methods).

^d^Current Behavior Algorithms.

ASD=autism spectrum disorder; T1=time 1; T2=time 2; FIQ=full-scale intelligence quotient; VIQ=verbal intelligence quotient; PIQ=performance intelligence quotient; M=male; F=female.

**Supplementary Table 4.** *Fit indices of the cross-lagged panel model.*

| *p*-value for model chi-square | 0.799 |
| --- | --- |
| Comparative fit index (CFI) | 1 |
| Tucker-Lewis Index (TLI) | 1.716 |
| Root mean square error of approximation (RMSEA) | 0.001 |
| Standardized root mean square residual (SRMS) | 0.048 |

**Supplementary Table 5.** *Post-hoc Sex-stratified pairwise t-tests on connectivity strength in the subnetwork showing a significant time by diagnosis interaction.*

| **Sex** | **Connectivity strength**  mean (standard deviation) | | **Post-hoc pairwise t-test statistics** | **Notes** |
| --- | --- | --- | --- | --- |
|  | **Time 1** | **Time 2** |  |  |
| **Autism spectrum disorder (ASD)** | | | | |
| **Male** (N=25) | 56.03 (36.92) | 38.29 (26.18) | *p*=0.001 | 5 male ASD showed the opposite direction from the group mean. |
| **Female** (N=2) | 49.49 | 34.14 | n.a. | 1 female ASD had increased connectivity; whereas the other showed reduced connections.  *The chi-square statistics for the sex ratio of the individuals showing the same direction of connection changes as the group mean: *p*=0.326. |
| **Typically developing control (TDC)** | | | | |
| **Male** (N=17) | 30.56 (19.88) | 40.12 (25.97) | *p*=0.015 | 6 male TDC showed the opposite direction from the group mean. |
| **Female** (N=12) | 27.38 (20.94) | 33.92 (28.27) | *p*=0.210 | 4 female TDC showed the opposite direction from the group mean.  *The chi-square statistics for the sex ratio of the individuals showing the same direction of connection changes as the group mean: *p*=0.913. |

**Supplementary Figure**

**Supplementary Figure 1.** *Symptom changes in each domain among youths with autism.* Panels depict changes in autism symptoms in each domain, as represented by scores on Current Behavior Algorithms of the Autism Diagnostic Interview-Revised (ADI-R), over time. For the male participants, the blue color denotes an improvement in symptoms; the red color indicates deteriorating symptoms over time; the green color represents unchanged autistic severity between the two assessments. The black color indicates female participants.


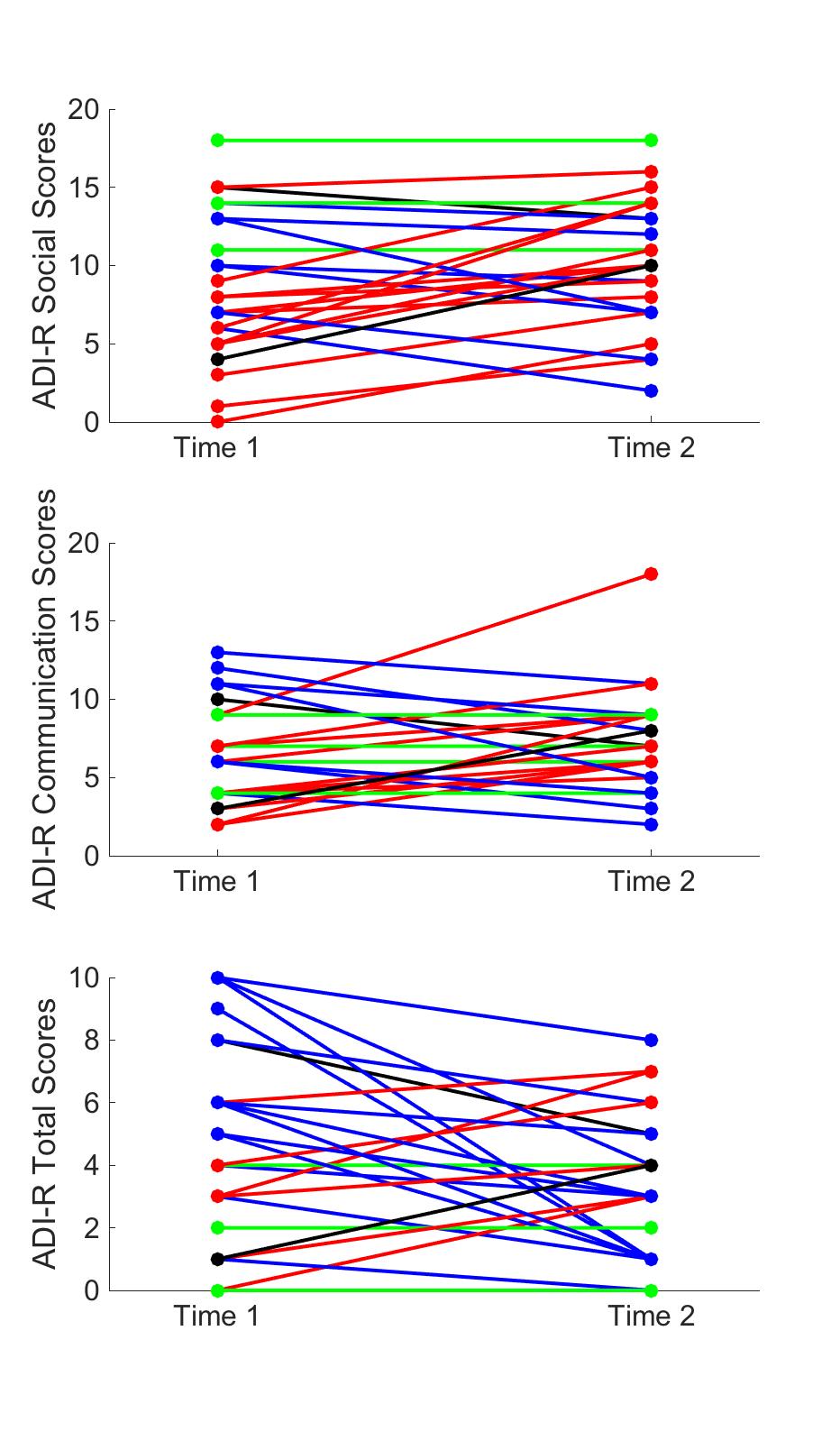


**Supplementary Figure 2.** *Distribution of streamline weights of subnetworks exhibiting a main effect of time.* Blue asterisks denote mean, while blue bars correspond to standard error of group-wise streamline weights. ASD=autism spectrum disorder; TDC=typically developing control.


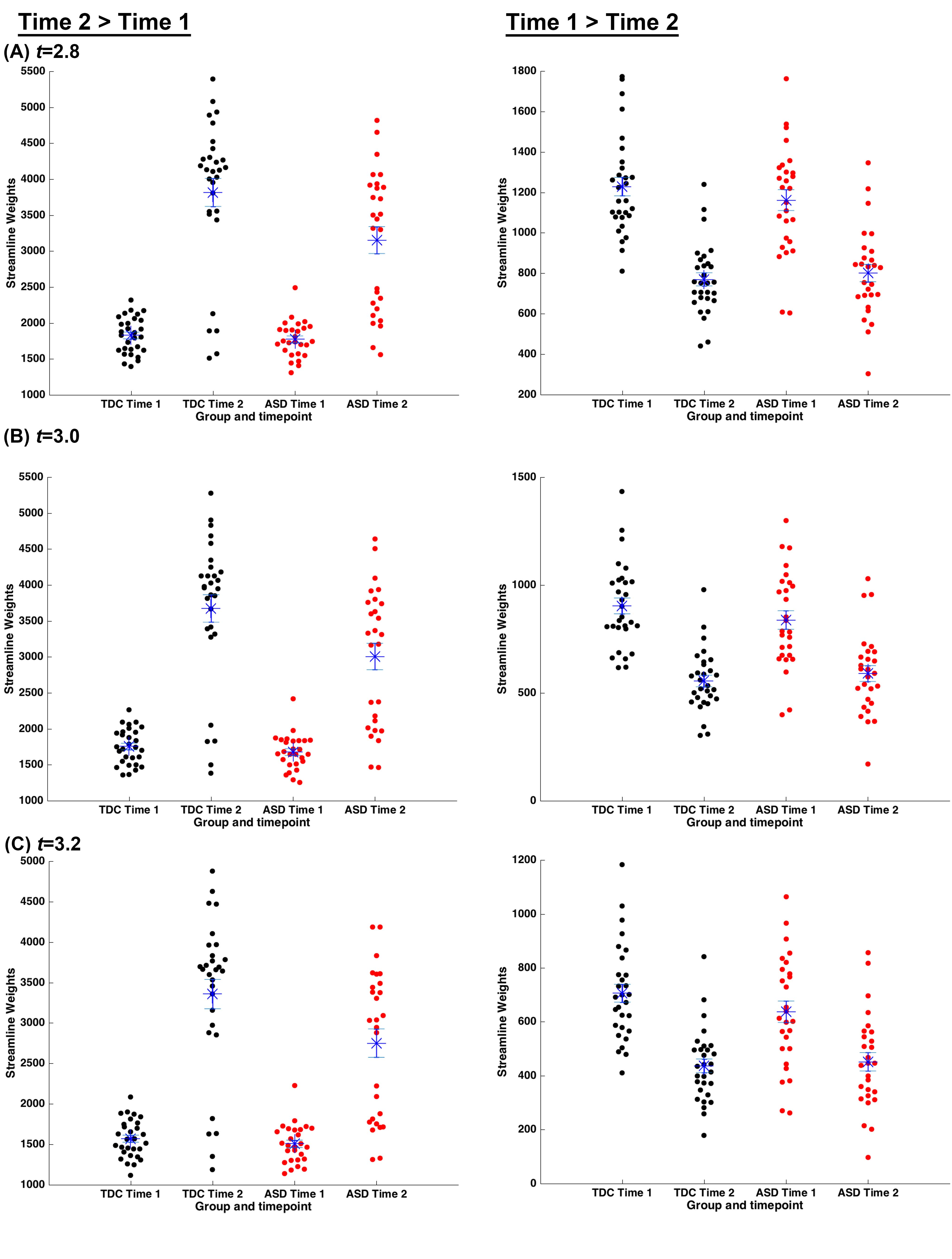


**Supplementary Figure 3.** *Subnetworks are exhibiting the main effects of time-based on a height threshold (t=2.8) in the network-based statistic.*


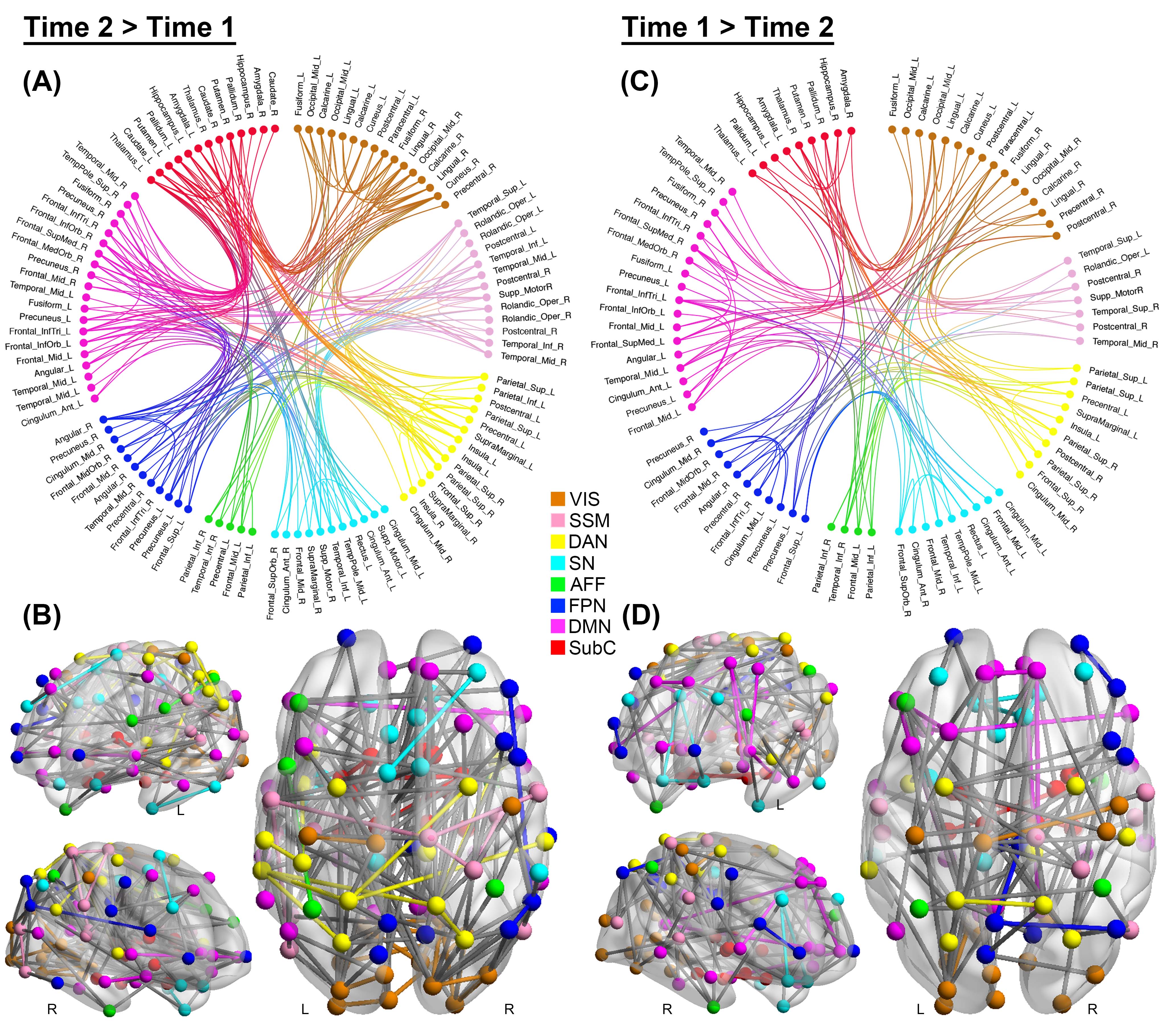


**Supplementary Figure 4.** *Subnetworks exhibiting the main effects of time-based on a height threshold (t=3.2) in the network-based statistic.*


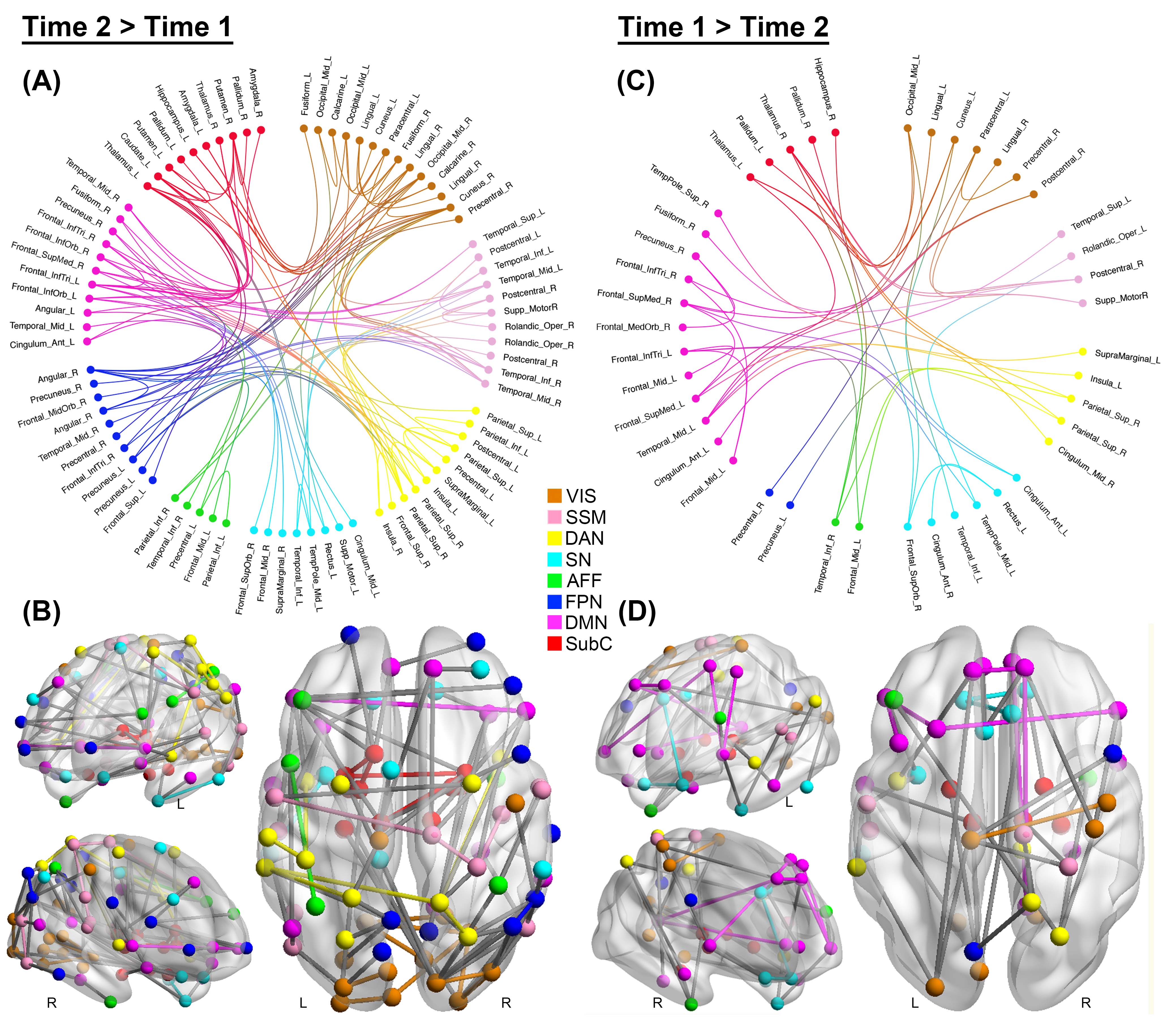


**Supplementary Figure 5.** *The subnetwork exhibiting a significant effect of time by diagnosis based on a height threshold (t=2.8) in the network-based statistic.*


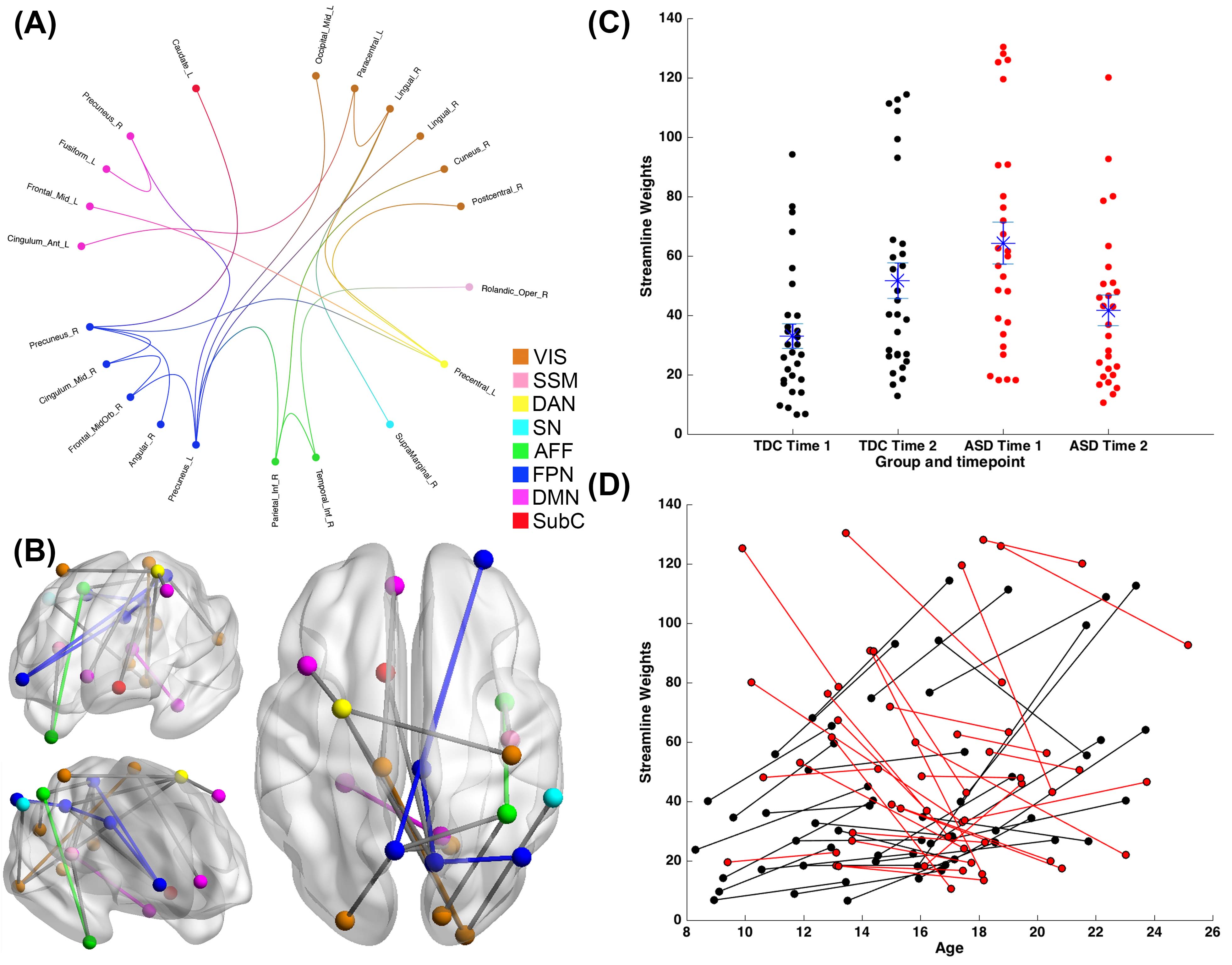


**Supplementary Figure 6.** *The subnetwork exhibiting a significant effect of time by diagnosis based on a height threshold (t=3.2) in the network-based statistic.*

*
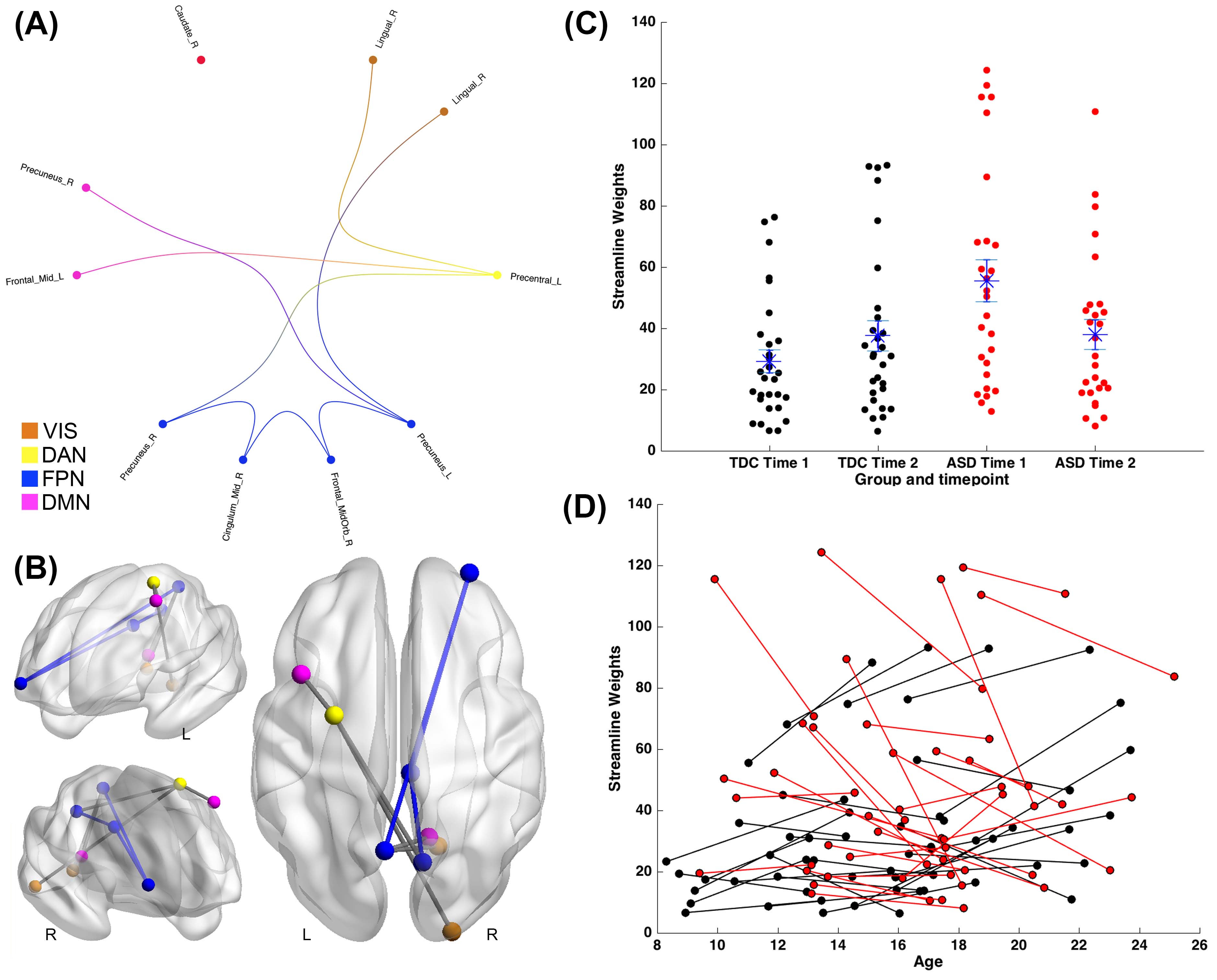
*

**Supplementary Figure 7.** *The subnetwork exhibiting a significant effect of time by diagnosis based on an auxiliary network-based statistic including an additional categorical nuisance covariate denoting comorbid and medication exposure status.*

*
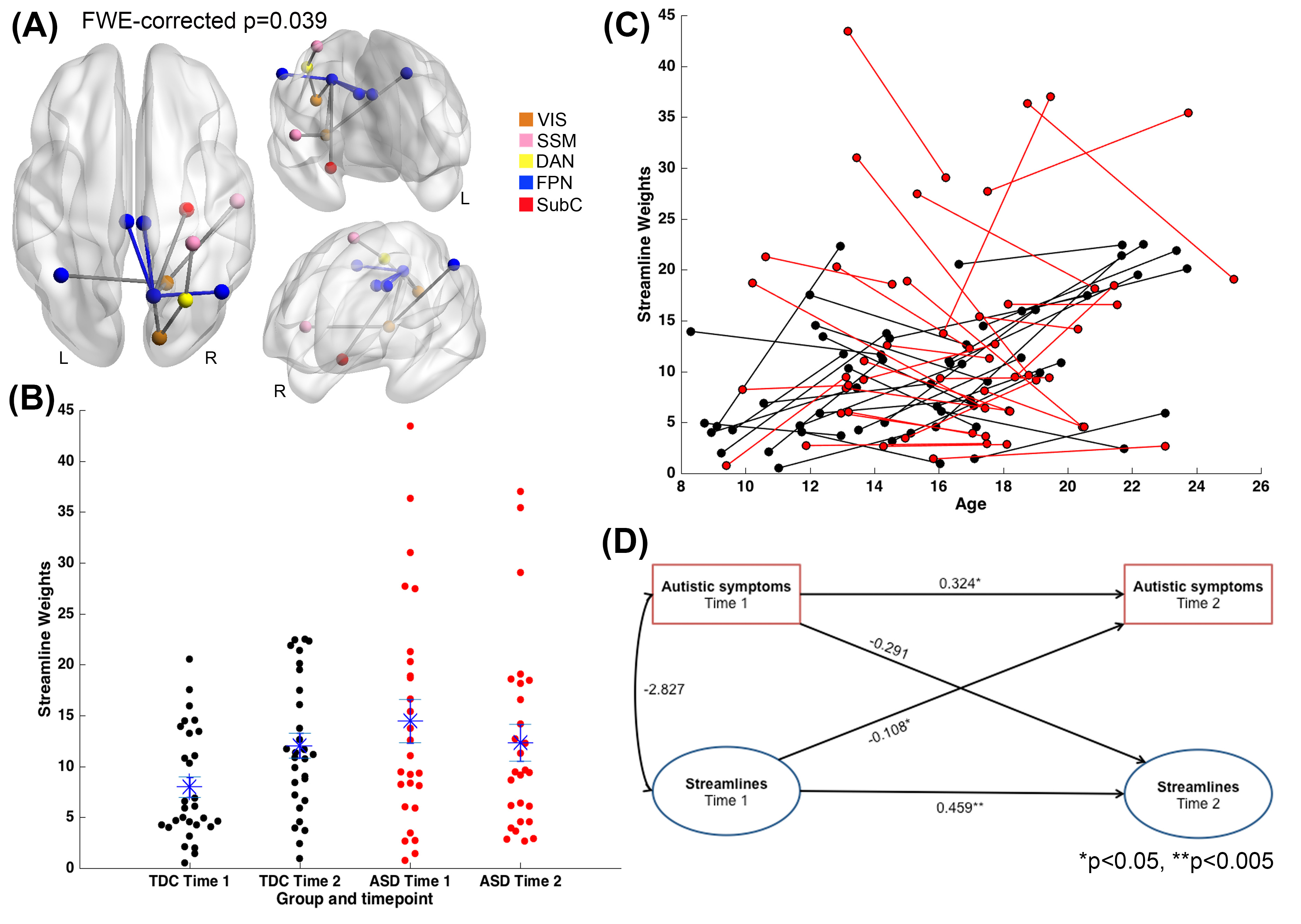
*
